# Successful resolution of basket impaction in pancreatic stone extraction using endoscopic papillary large balloon dilation

**DOI:** 10.1055/a-2663-8667

**Published:** 2025-08-14

**Authors:** Momoko Iketani, Kosuke Iwano, Shujiro Yazumi

**Affiliations:** 1 13867 Department of Gastroenterology and Hepatology, Kitano Hospital, Osaka, Japan

Basket impaction is a significant complication of pancreatic stone extraction. There have been a few reports highlighting the usefulness of endoscopic papillary large balloon dilation (EPLBD) in the pancreatic duct for managing large pancreatic stones 1 2 . Here we report a case of successful resolution of basket impaction during pancreatic stone extraction using EPLBD.

A 65-year-old man with chronic alcoholic pancreatitis underwent repeated endoscopic pancreatic stenting (EPS) for pancreatic duct stenosis and pancreatic stone extraction. Before admission, contrast-enhanced computed tomography revealed dilatation of the main pancreatic duct (MPD) and multiple pancreatic stones around the EPS ( Fig. 1 ).

**Fig. 1:**
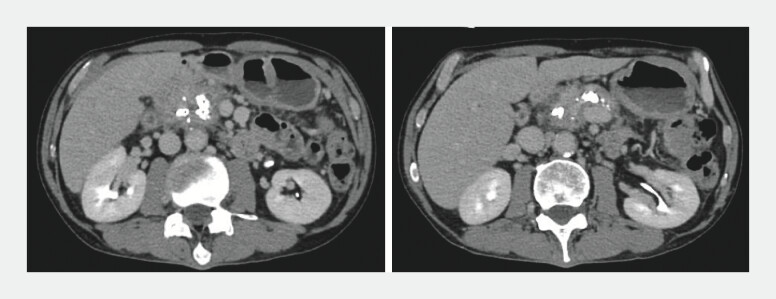
Contrast-enhanced computed tomography revealed the dilatation of the main pancreatic duct and multiple pancreatic stones around the endoscopic pancreatic stenting.

Endoscopic retrograde cholangiopancreatography was performed as a substitute for EPS. After EPS removal, pancreatography revealed multiple defects in the dilated MPD without significant stenosis. As these defects were suspected to represent protein plugs and small stones, stone extraction was performed using an eight-wire basket catheter (Medi-Globe 8-Wire Nitinol Basket; Medico’s Hirata Inc., Osaka, Japan). However, an unexpectedly large number of stones were captured, leading to basket impaction at the papilla ( Fig. 2 ).

**Fig. 2:**
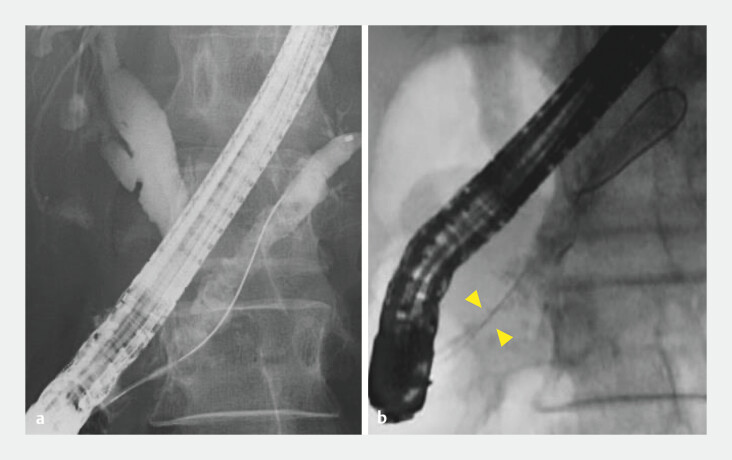
**a** Endoscopic retrograde pancreatography revealed multiple defects in the main pancreatic duct. **b** Using an eight-wire basket catheter, an unexpectedly large number of stones were captured, leading to basket impaction at the papilla (yellow arrowhead).

To resolve the impaction, the basket catheter wire was cut outside the scope, and the outer sheath was removed prior to performing EPLBD adjacent to the basket with a balloon catheter (Giga II balloon, 10–12 mm; Century Medical Inc., Tokyo, Japan). The balloon was inflated to a pressure of 4 atm (12 mm) and maintained for 1 min. During balloon deflation, the basket catheter was gently withdrawn, with successful retrieval of the impacted basket and stones without procedure-related complications ( Fig. 3 , Video 1 ).

**Fig. 3:**
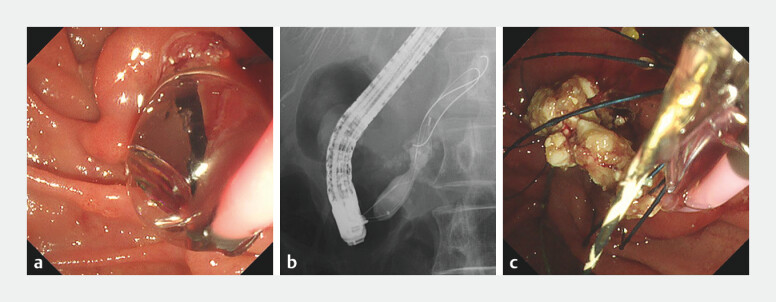
**a, b** Endoscopic papillary large balloon dilation was performed adjacent to the basket using a balloon catheter (Giga II balloon, 10–12 mm; Century Medical Inc., Tokyo, Japan). **c** During balloon deflation, the basket was gently withdrawn, and the impacted basket and stones were successfully retrieved.

During pancreatic stone extraction, basket impaction occurred unexpectedly, which was successfully resolved using endoscopic papillary large balloon dilation.Video 1

Although further studies to validate EPLBD’s safety and efficacy for similar cases are warranted, this case demonstrates that in situations where the pancreatic duct is sufficiently dilated, EPLBD can serve as an effective rescue technique when basket impaction occurs during the pancreatic stone extraction.

Endoscopy_UCTN_Code_TTT_1AR_2AH

## Notes

Endoscopy E-Videos https://eref.thieme.de/e-videos
*E-Videos* is an open access online section of the journal *Endoscopy* , reporting on interesting cases and new techniques in gastroenterological endoscopy. All papers include a high-quality video and are published with a Creative Commons CC-BY license. Endoscopy E-Videos qualify for HINARI discounts and waivers and eligibility is automatically checked during the submission process. We grant 100% waivers to articles whose corresponding authors are based in Group A countries and 50% waivers to those who are based in Group B countries as classified by Research4Life (see: https://www.research4life.org/access/eligibility/ ). This section has its own submission website at https://mc.manuscriptcentral.com/e-videos .
